# Mutations of the Transporter Proteins GlpT and UhpT Confer Fosfomycin Resistance in *Staphylococcus aureus*

**DOI:** 10.3389/fmicb.2017.00914

**Published:** 2017-05-19

**Authors:** Su Xu, Zhuyingjie Fu, Ying Zhou, Yang Liu, Xiaogang Xu, Minggui Wang

**Affiliations:** ^1^Institute of Antibiotics, Huashan Hospital, Fudan UniversityShanghai, China; ^2^Key Laboratory of Clinical Pharmacology of Antibiotics, National Health and Family Planning CommissionShanghai, China

**Keywords:** *Staphylococcus aureus*, fosfomycin, resistance, membrane transporter, *glpT*, *uhpT*

## Abstract

With the increasing spread of methicillin-resistant *Staphylococcus aureus* worldwide, fosfomycin has begun to be used more often, either alone or in combination with other antibiotics, for treating methicillin-resistant *S. aureus* infections, resulting in the emergence of fosfomycin-resistant strains. Fosfomycin resistance is reported to be mediated by fosfomycin-modifying enzymes (FosA, FosB, FosC, and FosX) and mutations of the target enzyme MurA or the membrane transporter proteins UhpT and GlpT. Our previous studies indicated that the *fos* genes might not the major fosfomycin resistance mechanism in *S. aureus*, whereas mutations of *glpT* and *uhpT* seemed to be more related to fosfomycin resistance. However, the precise role of these two genes in *S. aureus* fosfomycin resistance remains unclear. The aim of the present study was to investigate the role of *glpT* and *uhpT* in *S. aureus* fosfomycin resistance. Homologous recombination was used to knockout the *uhpT* and *glpT* genes in *S. aureus* Newman. Gene complementation was generated by the plasmid pRB473 carrying these two genes. The fosfomycin minimal inhibitory concentration (MIC) of the strains was measured by the *E*-test to observe the influence of gene deletion on antibiotic susceptibility. In addition, growth curves were constructed to determine whether the mutations have a significant influence on bacterial growth. Deletion of *uhpT, glpT*, and both of them led to increased fosfomycin MIC 0.5 μg/ml to 32 μg/ml, 4 μg/ml, and >1024 μg/ml, respectively. By complementing *uhpT* and *glpT* into the deletion mutants, the fosfomycin MIC decreased from 32 to 0.5 μg/ml and from 4 to 0.25 μg/ml, respectively. Moreover, the transporter gene-deleted strains showed no obvious difference in growth curves compared to the parental strain. In summary, our study strongly suggests that mutations of *uhpT* and *glpT* lead to fosfomycin resistance in *S. aureus*, and that *uhpT* mutation may play a more important role. The high resistance and low biological fitness cost resulting from *uhpT* and *glpT* deletion suggest that these strains might have an evolutionary advantage in a fosfomycin-rich clinical situation, which should be closely monitored.

## Introduction

*Staphylococcus aureus* is one of the most common bacterial pathogens worldwide in both community and hospital settings. Methicillin-resistant *Staphylococcus aureus* (MRSA) is an important multi-resistant pathogen. To date, vancomycin has remained the cornerstone drug in the management of invasive MRSA infections. However, the continuous rise in the vancomycin minimum inhibitory concentration (MIC), known as the “vancomycin MIC creep” phenomenon, poses a significant challenge to MRSA therapy; therefore, fosfomycin has recently been used alone or in combination with other antibiotics in treating MRSA infections (del Rio et al., 2016; VanEperen and Segreti, 2016). Nevertheless, this situation has inevitably led to the emergence of fosfomycin-resistant MRSA strains. In a recent review, the susceptibility of *S. aureus* to fosfomycin ranged between 33.2 and 100% in the nine available studies (frequency = 91.7%, 95% confidence interval 88.7–94.9%); in seven of the studies susceptibility rate was >90% (Vardakas et al., 2016). According to the CHINET surveillance program in China in 2010, 29.5% of the MRSA clinical isolates were resistant to fosfomycin (Guo et al., 2013). And Yu et al. (2010) reported a fosfomycin susceptible rate of 33.2%.

The mechanisms of action and resistance of fosfomycin have been studied for decades. Fosfomycin was first discovered in 1969 as an effective bactericidal agent against Gram-positive and Gram-negative organisms. The mechanism of action of fosfomycin differs from that of most commonly used antimicrobials. In general, fosfomycin is transported across the bacterial wall primarily with the help of the glycerol-3-phosphate (G-3-P) transport (GlpT) system. In the presence of glucose-6-phosphate (G-6-P), the hexose phosphate uptake transport (UhpT) system is induced, and provides an alternative route to the GlpT system. UhpT are important membrane transporter proteins for small molecules, including fosfomycin (Castaneda-Garcia et al., 2009). When transported into the cytosol of a bacterium, fosfomycin deactivates the target protein UDP-*N*-acetylglucosamine-3-enolpyruvyltransferase (MurA, encoded by the *murA* gene), thereby preventing the formation of *N*-acetylmuramic acid from *N*-acetylglucosamine and phosphoenolpyruvate, which is the initial step in peptidoglycan chain formation of the bacterial wall (Kahan et al., 1974). The key resistance mechanisms to fosfomycin include the loss or reduced production of transporters, reduced affinity to MurA, and production of fosfomycin-modifying enzymes (Sastry and Doi, 2016).

However, to date, the mechanisms contributing to fosfomycin resistance have been mostly studied in Gram-negative bacteria, with few related studies on Gram-positive bacteria. We have conducted several studies to investigate the fosfomycin resistance mechanisms in Gram-positive cocci, including *Enterococcus faecium* and *S. aureus* (Xu et al., 2013; Chen et al., 2014; Fu et al., 2016a,b). These previous studies indicated that the *fos* gene was not the major mechanism of fosfomycin resistance in MRSA isolates from our hospital, whereas mutations of *glpT* and *uhpT* seemed to be more closely related to fosfomycin resistance. However, the exact roles of these two genes in *S. aureus* fosfomycin resistance remain unclear. Thus, we designed the present study to investigate the roles of *glpT* and *uhpT* in *S. aureus* fosfomycin resistance.

## Materials and Methods

### Bacterial Strains and Plasmids

The strains and plasmids used in this study are presented in **Table 1**. The clinical fosfomycin-resistant MRSA strains SA2, SA94, and SA30 were collected from the blood or cerebral spinal fluid of patients at Huashan Hospital and were characterized previously (Fu et al., 2016b). And the strain names are in accordance with that in the **Supplementary Table S1** of the previous article (Fu et al., 2016b). Each of the clinical MRSA strains was with a different type of transporter gene mutation (**Table 1**). The *S. aureus* strains Newman and RN4220, and the plasmid pKOR1 were used in the homologous recombination assay (Bae and Schneewind, 2005; Wang et al., 2015). In addition, *S. aureus* ATCC29213 (American Type Tissue Culture Collection, Manassas, VA, United States) was used for the quality control of susceptibility testing. These strains and plasmids were laboratory collection.

**Table 1:** Strains and plasmids used to make the deletion mutations.

Strain, plasmid, or primer	Description	Source
***S. aureus* strains**		
SA2	MRSA carrying mutation on *glpT* and *uhpT*, fosfomycin MIC > 1024 μg/ml	Clinical strain 2 (Fu et al., 2016b)
SA94	MRSA carrying mutation on *uhpT*, fosfomycin MIC = 256 μg/ml	Clinical strain 94 (Fu et al., 2016b)
SA30	MRSA carrying mutation on *glpT*, fosfomycin MIC = 128 μg/ml	Clinical strain 30 (Fu et al., 2016b)
Newman	A fosfomycin-sensitive *S. aureus* strain, fosfomycin MIC = 0.5 μg/ml	Bae and Schneewind, 2005
RN4220	A non-a-hemolytic, non-restricting strain of *S. aureus*	Bae and Schneewind, 2005
Newman-*ΔuhpT*	*S. aureus* Newman with deletion of *uhpT*	This study
Newman-*ΔglpT*	*S. aureus* Newman with deletion of *glpT*	This study
Newman-*ΔuhpT*&*glpT*	*S. aureus* Newman with deletion of both *uhpT* and *glpT*	This study
Newman-*ΔuhpT*+pRB473-*uhpT*	Newman-*ΔuhpT* complemented with *uhpT* by plasmid pRB473	This study
Newman-*ΔglpT*+pRB473-*glpT*	Newman-*ΔglpT* complemented with *glpT* by plasmid pRB473	This study
SA2+pRB473-*uhpT*	SA2 complemented with *uhpT* by plasmid pRB473	This study
SA2+pRB473-*glpT*	SA2 complemented with *glpT* by plasmid pRB473	This study
SA94+pRB473-*uhpT*	SA94 complemented with *uhpT* by plasmid pRB473	This study
SA94+pRB473-*glpT*	SA94 complemented with *glpT* by plasmid pRB473	
SA30+pRB473-*uhpT*	SA30 complemented with *uhpT* by plasmid pRB473	
SA30+pRB473-*glpT*	SA30 complemented with *glpT* by plasmid pRB473	This study
**Plasmids**		
pKOR1	*E. coli* – *S. aureus* shuttle vector; Amp^r^ in *E. coli*; Cm^r^ in *S. aureus*	Bae and Schneewind, 2005
pKOR1-*ΔuhpT*	pKOR1 with deletion mutation of *uhpT*	This study
pKOR1-*ΔglpT*	pKOR1 with deletion mutation of *glpT*	This study
pRB473	*E. coli* – *S. aureus* shuttle vector; Cm^r^ in *S. aureus*	Wang et al., 2015
pRB473-*uhpT*	pRB473 ligated with *uhpT*	This study
pRB473-*glpT*	pRB473 ligated with *glpT*	This study

### Allelic Gene Deletion by Homologous Recombination

Knockout of the transporter genes *glpT* and *uhpT* was conducted as previously described (Bae and Schneewind, 2005; Wang et al., 2015). The plasmids and primers used are listed in **Tables 1, 2**, respectively. Proper gene deletion was verified by analytical polymerase chain reaction (PCR) and sequencing of the genomic DNA at the borders of the PCR-derived regions. Sequencing was then performed to confirm the nucleotides. The amplified fragments were used to construct the homologous recombinant pKOR1-*ΔuhpT/glpT* with Gateway^®^BP Clonase^TM^ II Enzyme mix (Thermo Fisher Scientific, Waltham, MA, United States).

**Table 2:** Primers for PCR and sequencing.

Primers	Sequence (5′–3′)	Application
attB1-uhpT-up-F	ggggacaagtttgtacaaaaaagcaggctAAATGCCTCTACACCAG	Allelic replacement
uhpT-NR-EcoRI	CCGgaattcTTGTTCGGAATCTTATGG	
attB2-uhpT-CF	ggggaccactttgtacaagaaagctgggtAATTGCAGACAAAGTAGG	
uhpT-CR-EcoRI	CCGgaattcTCTATGTTGCATTATTCCTA	
attB1-glpT-up-F	ggggacaagtttgtacaaaaaagcaggctATCGGCGTTATCTTTGTTG	
glpT-NR-EcoRI	CCGgaattcGGATGGGATGTCGGTTT	
attB2-glpT-CF	ggggaccactttgtacaagaaagctgggtAACCTTGTGGTGCTAATGTC	
glpT-CR-EcoRI	CCGgaattcCAGCGTAACCGATGAAAAT	
C-uhpT-F	CGCggatccGATTATTGTAAGCAAGCAA	Construction of complemented strain
C-uhpT-R	CCGgaattcTAACGCCATATTCAACTG	
C-glpT-F	CGCggatccTTAATGATGAACAGTTTCTT	
C-glpT-R	CGGggtaccTATTCATACTATCCCTCCT	

pKOR1-*ΔuhpT* and pKOR1-*ΔglpT* were introduced into *S. aureus* RN4220 by electroporation for modification. The plasmid extracted from strain RN4220 was then introduced into *S. aureus* Newman. The desired *uhpT* and *glpT* deletion mutants were selected as described previously (Bae and Schneewind, 2005).

The successful generation of the Newman-*ΔuhpT* and Newman-*ΔglpT* strains was further confirmed by PCR and sequencing. PCRs were performed using the primers attB1-*uhpT*-up-F/attB2-*uhpT*-CF and attB1-*glpT*-up-F/attB2-*glpT*-CF in the strains *S. aureus* Newman, Newman-*ΔuhpT*, and Newman-*ΔglpT*, respectively.

### Construction of the Complemented Strain

Fragments were PCR-amplified from *S. aureus* Newman using the primers C-*uhpT*-F/R and C-*glpT*-F/R. The PCR products and vector pRB473 were double-digested with the designed restriction enzymes BamHI and EcoRI (for *uhpT*), or BamHI and KpnI (for *glpT*), and ligation was performed with T4 ligase. The resulting plasmids were transferred into *S. aureus* RN4220, and then introduced into the deletion and clinical strains with defects on *uhpT* and/or *glpT*, SA2, SA94, and SA30.

### Antimicrobial Susceptibility Testing

Fosfomycin susceptibility of the knockout and clinical strains with defects on *uhpT* and/or *glpT*, and their complemented strains were tested with the *E*-test (BioMerieux SA, La Balme Les Grotts, France), according to the manufacturer’s guidance. Results were interpreted according to European committee on antimicrobial susceptibility testing criteria (European Committee on Antimicrobial Susceptibility Testing [EUCAST], 2017) (susceptible, ≤32 mg/L; resistant, ≥64 mg/L).

### Measurement of Growth Curves

To evaluate the influence of deletion of the transporter genes on bacterial growth, we measured the *in vitro* growth curves of *S. aureus* Newman, Newman-*ΔuhpT*, Newman-*ΔglpT*, Newman-*ΔuhpT*&*glpT*, and the clinical strains. The strains were cultivated in tryptic soy broth overnight at 37°C. The bacterial solution was diluted to an optical density at 600 nm (OD_600_) of 0.1 and cultivated again. The OD_600_ was then measured at 0, 2, 4, 6, 8, 10, 12, 14, 16, 18, 20, 22, and 24 h for each strain, by spectrophotometer (UNICO, Shanghai, China). The procedure was repeated three times for each strain, and the mean OD_600_ values were used to draw the growth curves.

### Phenotype Microarray (PM) Analysis

Phenotype Microarray analysis was performed using BIOLOG Phenotype Microarray^TM^ (BIOLOG, Hayward, CA, United States) according to the manufacturer’s recommendations. The deletion mutants, namely Newman-*ΔuhpT*, Newman-*ΔglpT*, Newman-*ΔuhpT*&*glpT*, and the parental strain Newman, were tested with the 96-wells plates PM1 and PM2, containing 190 carbon substrates, including G-6-P (PM1 plate, well C1). To assess the altered phenotypes in carbon metabolism of the deletion mutants, the growth was compared to the parent *S. aureus* Newman.

## Results

The deletion mutants showed considerably increased MIC values to fosfomycin compared to that of the parental strain. The Newman-*ΔuhpT*&*glpT* strain, in which both transporter genes were knocked out, showed high-level resistance (MIC > 1024 μg/ml) to fosfomycin, as determined by the *E*-test (**Table 3**). When *uhpT* or *glpT* was knocked out from *S. aureus* Newman, the fosfomycin MICs increased from 0.5 to 32 μg/ml or 4 μg/ml, respectively.

**Table 3:** Fosfomycin MIC (μg/ml) of *S. aureus* mutant strains and complemented strains.

*S. aureus* strains	Fosfomycin MIC
Newman-*ΔglpT*	4
Newman-*ΔglpT*+pRB473-*glpT*	0.25
Newman-*ΔuhpT*	32
Newman-*ΔuhpT*+pRB473-*uhpT*	0.5
Newman-*ΔuhpT*&*glpT*	>1024
SA2	>1024
SA2+pRB473-*glpT*	>1024
SA2+pRB473-*uhpT*	16
SA94	256
SA94+pRB473-*glpT*	256
SA94+pRB473-*uhpT*	16
SA30	128
SA30+pRB473-*glpT*	32
SA30+pRB473-uhpT	64

Complementing *uhpT* and *glpT* led to a reduced fosfomycin MIC in the deletion mutants and the clinical fosfomycin-resistant *S. aureus* strains with defects at both sites. By complementing plasmid pRB473-*uhpT* into Newman-*ΔuhpT*, the strain’s fosfomycin MIC decreased from 32 to 0.5 μg/ml. Similarly, by complementing *glpT* into Newman-*ΔglpT*, strain’s fosfomycin MIC decreased from 4 to 0.25 μg/ml. *S. aureus* SA2, SA94, and SA30 were clinical fosfomycin-resistant strains, with mutations of both *uhpT* and *glpT, uhpT* only, and *glpT* only, respectively. When complemented with the functional transporter genes, the fosfomycin MICs decreased considerably, as shown in **Table 3**.

**FIGURE 1:**
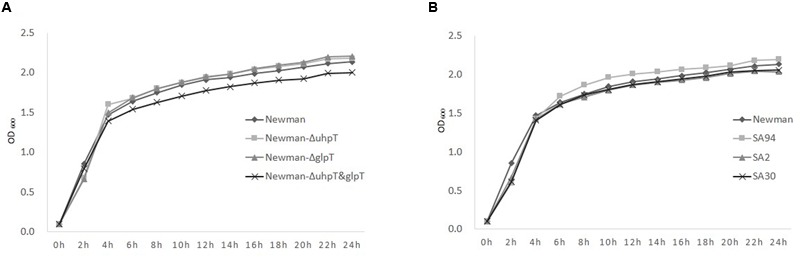
**The *in vitro* growth curves of *S. aureus* strains.** The strains were cultivated in tryptic soy broth overnight at 37°C. The bacterial solution was diluted to an optical density at 600 nm (OD_600_) of 0.1 and cultivated again. The OD_600_ was then measured at 0, 2, 4, 6, 8, 10, 12, 14, 16, 18, 20, 22, and 24 h to draw the curves. **(A)** Newman (◆), Newman-*ΔuhpT* (■), Newman-*ΔglpT* (▲), and Newman-*ΔuhpT*&*glpT* (×). **(B)** Newman (◆) and the clinical *uhpT/glpT* mutants: SA2 (▲), with mutations of both *glpT* and *uhpT*; SA94 (■), with mutation of *uhpT*; SA30 (×), with mutation of *glpT*.

*In vitro* bacterial growth curves of the wild-type strain *S. aureus* Newman and the deletion mutants were compared to evaluate the potential fitness cost of these resistant-conferring mutations. As shown in **Figure 1**, no significant depression in growth was observed in Newman-*ΔuhpT* and Newman-*ΔglpT* compared to the wild-type strain. However, the strain Newman-*ΔuhpT*&*glpT* presented slight growth inhibition compared to the wild-type.

**FIGURE 2:**
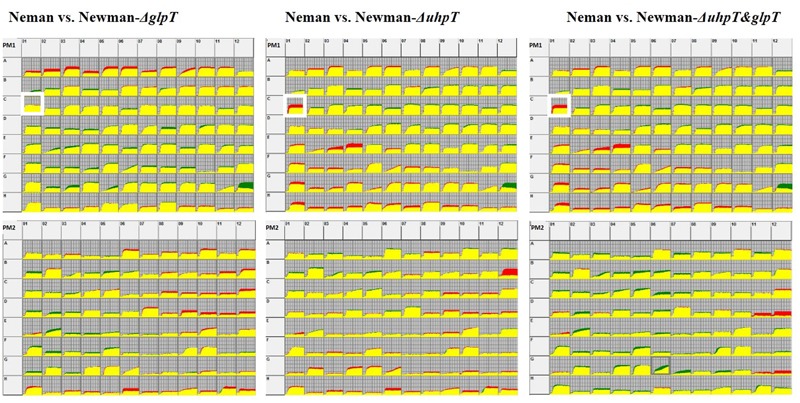
**PM analysis for carbon metabolism.** The strains were grown in a 96-well plate under 37°C over two nights. Carbon utilization kinetics was measured with OmniLog instrument. Data were superimposed using OmniLog software. The kinetic results show consensus data comparing *S. aureus* Newman and its transporter gene deletion mutants. A metabolic advantage by *S. aureus* Newman is indicated by red, while a metabolic advantage by the deletion mutants is shown by green. The yellow part indicates that both strains have equal metabolism in the well. The white box around PM1 plate, well C1, highlights the G-6-P metabolism. Detailed substrate information of PM1 and PM2 were shown in the **Supplementary Table S1**.

Phenotype Microarray analysis was performed using carbon utilization panels, PM1 and PM2, in 190 carbon substrates. The changes in carbon metabolism were listed in **Figure 2**. *S. aureus* Newman showed metabolic advantage over Newman-*ΔuhpT* and Newman-*ΔuhpT*&*glpT* in wells containing G-6-P (**Figure 2**, PM1, well C1). G-3-P was not included in the substrate list, and there was no obvious change found in Newman-*ΔglpT*.

## Discussion

Intravenous fosfomycin is broadly used in the treatment of multidrug-resistant pathogens in Europe and Asia owing to its unique antibiotic mechanism, high permeability, and high susceptibility rate (Falagas et al., 2009). Falagas et al. (2010) performed fosfomycin susceptibility testing in non-urinary MRSA isolates, among which 99.2% (129/130) were found to be susceptible. The same group reviewed the susceptibility data of Gram-positive cocci, and reported a cumulative susceptibility rate of 87.9% (4240/4892) in *S. aureus* (Falagas et al., 2009).

Fosfomycin-resistance mechanism has been well described for Gram-negative bacteria such as *E. coli* (Kim et al., 1996; Horii et al., 1999; Huang et al., 2003; Takahata et al., 2010). In *E. coli*, GlpT and UhpT are responsible for fosfomycin uptake. Mutations or insertional inactivation in the *glpT* and/or *uhpT* genes or their regulatory genes lead to the loss of function of the transporters and fosfomycin resistance. The inactivation of either *uhpT* or *glpT* conferred a moderate fosfomycin resistance, (MICs increased from 2 to 8 μg/ml and 32 μg/ml, respectively, compared to the wild type) (Takahata et al., 2010). In *P. aeruginosa*, the inactivation of *glpT* produced significant decrease in fosfomycin MIC, from 8 to 1024 μg/ml (Castaneda-Garcia et al., 2009; Takahata et al., 2010). Modification or overexpression of *murA*, production of fosfomycin-modifying enzymes, are also associated with fosfomycin resistance (Garcia et al., 1995; Kim et al., 1996; Bernat et al., 1997; Horii et al., 1999; Fillgrove et al., 2003; Roberts et al., 2013).

There is less known of fosfomycin resistance mechanism in Gram-positive cocci. In previous works, we collected MRSA clinical strains, and found that only the minority of the fosfomycin-resistant MRSA strains carried the fos gene or murA mutation, while glpT and uhpT mutations were common (82.1%, 55/67, vs. 77.6%, 52/67, respectively) (Fu et al., 2016a,b). This fact indicated that, fosB or murA mutation is not the major contributor to fosfomycin resistance in MRSA, while mutations within the glpT and/or uhpT genes might play an important role in *S. aureus* fosfomycin resistance. In the present study, we established uhpT and/or glpT deletion mutants. Knocking out both genes resulted in high-level fosfomycin resistance (MIC > 1024 μg/ml). Complementing either of the two genes into the deletion mutants and clinical mutated strains resulted in a decreased fosfomycin MIC. Direct comparison of uhpT and glpT according to the level of increase of the MIC suggested that uhpT has a greater effect on the strain’s MIC to fosfomycin than glpT.

To evaluate the possible, the *in vitro* fitness cost of the transporter gene mutation, we compared growth curves between the fosfomycin-sensitive wild-type strain, laboratory deletion mutant strain, and clinical strains with defects on *uhpT* and/or *glpT*. Previous reports have shown that mutations of *uhpT* and *glpT* can compromise the growth of strains of *E. coli, Klebsiella pneumoniae*, and *Proteus mirabilis* (Li Pira et al., 1987; Marchese et al., 2003). A probable mechanism might be that mutations of the *glpT* and/or *uhpT* transporting systems prevent carbon source getting into the cytoplasm, and therefore disturb cell metabolism (Venkateswaran and Wu, 1972; Nilsson et al., 2003). But in *P. aeruginosa*, the *glpT* mutation was found to lead to fosfomycin resistance with no obvious fitness cost (Castaneda-Garcia et al., 2009). In the present study, there was only a slight reduction of growth observed in the strain Newman-*ΔuhpT*&*glpT* compared to the wild type, and no significant growth suppression was observed either in the laboratory deletion mutant strains or in clinical strains with defects on *uhpT* and/or *glpT*, which is similar as observed in *P. aeruginosa*. So *S. aureus* might also compensate the disadvantage in energy obtainment caused by *uhpT* and/or *glpT* mutation through other transporting systems. But further study is still in need for verification.

We observed that G-6-P utilization was defected in both Newman-*ΔuhpT* and Newman-*ΔuhpT*&*glpT*. UhpT is the membrane transporter of this substrate, deletion mutants of *uhpT* showed defects in G-6-P metabolism is as expected. The G-3-P metabolism in *S. aureus* seems to be more complicated. G-3-P seems to be an intermediate product in carbon/phosphorus metabolism pathway. As another low G+C Gram-positive bacteria, *B. subtilis* shares similar carbon metabolism pattern as *S. aureus*. In *B. subtilis*, G-3-P is produced from glycerol with glycerol kinase. And G-3-P dehydrogenase can oxidize G-3-P to dihydroxyacetone phosphate, an intermediated in glycolysis (Holmberg et al., 1990). We have not observed significant change in metabolism. This may be because that G-3-P utilization defect is easily compensated by other pathways.

In summary, the results of our study strongly suggest that mutations of uhpT and glpT lead to fosfomycin resistance in *S. aureus*, and that the uhpT mutation may play a more important role. The high resistance and low fitness cost resulting from uhpT and glpT mutations suggest that these mutated strains might have an evolutionary advantage in a fosfomycin-rich clinical situation. The widely observed uhpT or glpT mutation in *S. aureus* might be a threat in hospital settings. Further studies are needed to evaluate the frequency of *S. aureus* fosfomycin mutants, and virulence of these mutants.

## Author Contributions

Designed and conceived the experiments: YL, XX, and MW. Performed the experiments: SX, ZF, and YZ. Analyzed the data: SX, ZF, and YZ. Wrote and reviewed the manuscript: SX, ZF, XX, and YL.

## Conflict of Interest Statement

The authors declare that the research was conducted in the absence of any commercial or financial relationships that could be construed as a potential conflict of interest.

## Supplementary Material

TABLE S1 **Carbon sources tested in PM1 and PM2 plates**.
