# Supplementary material for: Mutations of the Transporter Proteins GlpT and UhpT Confer Fosfomycin Resistance in Staphylococcus aureus
Source: Front Microbiol. 2017 May 19;8:914. doi: 10.3389/fmicb.2017.00914 (PMC5437707; doi:10.3389/fmicb.2017.00914)
Supplement: TABLE S1 — Carbon sources tested in PM1 and PM2 plates. [file Table_1.PDF]

BIOLOG

Phenotype MicroArrays™

PM1 MicroPlate™ Carbon Sources

A1<br>Negative Control	A2<br>L-Arabinose	A3<br>N-Acetyl-D-Glucosamine	A4<br>D-Saccharic Acid	A5<br>Succinic Acid	A6<br>D-Galactose	A7<br>L-Aspartic Acid	A8<br>L-Proline	A9<br>D-Alanine	A10<br>D-Trehalose	A11<br>D-Mannose	A12<br>Dulcitol
B1<br>D-Serine	B2<br>D-Sorbitol	B3<br>Glycerol	B4<br>L-Fucose	B5<br>D-Glucuronic Acid	B6<br>D-Gluconic Acid	B7<br>D,L-α-Glycerol-Phosphate	B8<br>D-Xylose	B9<br>L-Lactic Acid	B10<br>Formic Acid	B11<br>D-Mannitol	B12<br>L-Glutamic Acid
C1<br>D-Glucose-6-Phosphate	C2<br>D-Galactonic Acid-γ-Lactone	C3<br>D,L-Malic Acid	C4<br>D-Ribose	C5<br>Tween 20	C6<br>L-Rhamnose	C7<br>D-Fructose	C8<br>Acetic Acid	C9<br>α-D-Glucose	C10<br>Maltose	C11<br>D-Melibiose	C12<br>Thymidine
D-1<br>L-Asparagine	D2<br>D-Aspartic Acid	D3<br>D-Glucosaminic Acid	D4<br>1,2-Propanediol	D5<br>Tween 40	D6<br>α-Keto-Glutaric Acid	D7<br>α-Keto-Butyric Acid	D8<br>α-Methyl-D-Galactoside	D9<br>α-D-Lactose	D10<br>Lactulose	D11<br>Sucrose	D12<br>Uridine
E1<br>L-Glutamine	E2<br>M-Tartaric Acid	E3<br>D-Glucose-1-Phosphate	E4<br>D-Fructose-6-Phosphate	E5<br>Tween 80	E6<br>α-Hydroxy Glutaric Acid-γ-Lactone	E7<br>α-Hydroxy Butyric Acid	E8<br>β-Methyl-D-Glucoside	E9<br>Adonitol	E10<br>Maltotriose	E11<br>2-Deoxy Adenosine	E12<br>Adenosine
F1<br>Glycyl-L-Aspartic Acid	F2<br>Citric Acid	F3<br>M-Inositol	F4<br>D-Threonine	F5<br>Fumaric Acid	F6<br>Bromo Succinic Acid	F7<br>Propionic Acid	F8<br>Mucic Acid	F9<br>Glycolic Acid	F10<br>Glyoxylic Acid	F11<br>D-Cellobiose	F12<br>Inosine
G1<br>Glycyl-L-Glutamic Acid	G2<br>Tricarballylic Acid	G3<br>L-Serine	G4<br>L-Threonine	G5<br>L-Alanine	G6<br>L-Alanyl-Glycine	G7<br>Acetoacetic Acid	G8<br>N-Acetyl-β-D-Mannosamine	G9<br>Mono Methyl Succinate	G10<br>Methyl Pyruvate	G11<br>D-Malic Acid	G12<br>L-Malic Acid
H1<br>Glycyl-L-Proline	H2<br>p-Hydroxy Phenyl Acetic Acid	H3<br>m-Hydroxy Phenyl Acetic Acid	H4<br>Tyramine	H5<br>D-Psicose	H6<br>L-Lyxose	H7<br>Glucuronamide	H8<br>Pyruvic Acid	H9<br>L-Galactonic Acid-γ-Lactone	H10<br>D-Galacturonic Acid	H11<br>Phenylethyl-amine	H12<br>2-Aminoethanol

PM2A MicroPlate™ Carbon Sources

A1<br>Negative Control	A2<br>Chondroitin Sulfate C	A3<br>α-Cyclodextrin	A4<br>β-Cyclodextrin	A5<br>γ-Cyclodextrin	A6<br>Dextrin	A7<br>Gelatin	A8<br>Glycogen	A9<br>Inulin	A10<br>Laminarin	A11<br>Mannan	A12<br>Pectin
B1<br>N-Acetyl-D-Galactosamine	B2<br>N-Acetyl-Neuraminic Acid	B3<br>β-D-Allose	B4<br>Amygdalin	B5<br>D-Arabinose	B6<br>D-Arabitol	B7<br>L-Arabitol	B8<br>Arbutin	B9<br>2-Deoxy-D-Ribose	B10<br>I-Erythritol	B11<br>D-Fucose	B12<br>3-0-β-D-Galacto-pyranosyl-D-Arabinose
C1<br>Gentiobiose	C2<br>L-Glucose	C3<br>Lactitol	C4<br>D-Melezitose	C5<br>Maltitol	C6<br>α-Methyl-D-Glucoside	C7<br>β-Methyl-D-Galactoside	C8<br>3-Methyl Glucose	C9<br>β-Methyl-D-Glucuronic Acid	C10<br>α-Methyl-D-Mannoside	C11<br>β-Methyl-D-Xyloside	C12<br>Palatinose
D1<br>D-Raffinose	D2<br>Salicin	D3<br>Sedoheptulosan	D4<br>L-Sorbose	D5<br>Stachyose	D6<br>D-Tagatose	D7<br>Turanose	D8<br>Xylitol	D9<br>N-Acetyl-D-Glucosaminitol	D10<br>γ-Amino Butyric Acid	D11<br>δ-Amino Valeric Acid	D12<br>Butyric Acid
E1<br>Capric Acid	E2<br>Caproic Acid	E3<br>Citraconic Acid	E4<br>Citramalic Acid	E5<br>D-Glucosamine	E6<br>2-Hydroxy Benzoic Acid	E7<br>4-Hydroxy Benzoic Acid	E8<br>β-Hydroxy Butyric Acid	E9<br>γ-Hydroxy Butyric Acid	E10<br>α-Keto Valeric Acid	E11<br>Itaconic Acid	E12<br>5-Keto-D-Gluconic Acid
F1<br>D-Lactic Acid Methyl Ester	F2<br>Malonic Acid	F3<br>Melibionic Acid	F4<br>Oxalic Acid	F5<br>Oxalomalic Acid	F6<br>Quinic Acid	F7<br>D-Ribono-1,4-Lactone	F8<br>Sebacic Acid	F9<br>Sorbic Acid	F10<br>Succinamic Acid	F11<br>D-Tartaric Acid	F12<br>L-Tartaric Acid
G1<br>Acetamide	G2<br>L-Alaninamide	G3<br>N-Acetyl-L-Glutamic Acid	G4<br>L-Arginine	G5<br>Glycine	G6<br>L-Histidine	G7<br>L-Homoserine	G8<br>Hydroxy-L-Proline	G9<br>L-Isoleucine	G10<br>L-Leucine	G11<br>L-Lysine	G12<br>L-Methionine
H1<br>L-Ornithine	H2<br>L-Phenylalanine	H3<br>L-Pyroglutamic Acid	H4<br>L-Valine	H5<br>D,L-Carnitine	H6<br>Sec-Butylamine	H7<br>D.L-Octopamine	H8<br>Putrescine	H9<br>Dihydroxy Acetone	H10<br>2,3-Butanediol	H11<br>2,3-Butanone	H12<br>3-Hydroxy 2-Butanone
